# A new species of *Argyreia* (Convolvulaceae) from Thailand

**DOI:** 10.3897/phytokeys.149.50379

**Published:** 2020-06-04

**Authors:** Paweena Traiperm, Somran Suddee

**Affiliations:** 1 Department of Plant Science, Faculty of Science, Mahidol University, Rama VI Road, Ratchathewi, Bangkok 10400, Thailand Mahidol University Bangkok Thailand; 2 Forest Herbarium, Department of National Parks, Wildlife and Plant Conservation, Chatuchak, Bangkok 10900, Thailand Wildlife and Plant Conservation Bangkok Thailand

**Keywords:** filament morphology, new species discovery, Phu Langka, SE Asian biodiversity, staminal trichomes, taxonomy

## Abstract

*Argyreia pseudosolanum* Traiperm & Suddee, **sp. nov.** from the NE region of Thailand is described and illustrated. The new species is remarkable in having a very distinctive corolla shape similar to *Solanum*, and staminal filament bases glabrous or nearly glabrous with a few multicellular, uniseriate hairs at the attachment point on the corolla tube. Detailed descriptions, illustrations, a summary of the ecology and an IUCN conservation status are provided.

## Introduction

*Argyreia* Lour. is one of the larger genera in the spiny pollen group of Convolvulaceae, and is well characterised by indehiscent fleshy or mealy berries (Staples and Traiperm 2017). The genus is distributed mainly in tropical Asia (Staples and Traiperm 2017), except for four species that are geographically disjunct from Asia and located in Madagascar (Deroin 2001).

A checklist of the genus *Argyreia* Lour. (Convolvulaceae) has been recently published reporting 135 species and 5 varieties in total (Staples and Traiperm 2017). However, the number of species has been actively increasing and is now up to 140 species due to the fact that two novelties have been discovered in Myanmar (Traiperm et al. 2019) and Thailand (Chitchak et al. 2018), as well as a transfer of *Ipomoea nana* Collett & Hemsl. into *Argyreia* (Shalini et al. 2017). Since the account of Thai Convolvulaceae was published (Staples and Traiperm 2010), six more new Thai species of *Argyreia* have been named to date (Chitchak et al. 2018; Staples et al. 2015; Traiperm and Staples 2014, 2016). Up to now, Thailand has 40 species in total (the highest number of species for any country studied so far in tropical Asia) or approximately one-third of all species, making the country a centre of species richness in *Argyreia*. However, the identification of some species in Thailand is still inconclusive, because the specimens of these species lack reproductive organs, either partially or entirely.

All species of the genus *Argyreia* have recently been transferred to *Ipomoea* L. by Muñoz-Rodríguez et al. (2019). We chose, however, not to follow the proposed classification in the present work, as further study of Old World taxa is still required.

During field surveys in Phu Langka National Park, Bueng Kan Province (NE Thailand) by the second author, an unrecognised *Argyreia* species was discovered. The plant was distinctive for its unusual corolla shape, similar to those found in the family Solanaceae. The specimens were then compared with the type specimens of other *Argyreia* species that have similar corolla shapes at the three main herbaria in Thailand (BK, BKF and QBG), as well as three major herbaria in the UK (BM, K and K-W) by the first author. This is one of several unknown *Argyreia* that could not be linked to any previously published names. We therefore describe and illustrate a new *Argyreia* species from Thailand here.

## Materials and methods

Plant materials were collected from Phu Langka National Park in 2018. Morphological measurements were made from dried herbarium specimens. The collected specimens were compared with the type specimens of morphologically similar species at BK, BKF, BM, K, K-W and QBG herbaria, as well as digital images available online from other herbaria (both via JSTOR and the following online collections: G, P, L and NY). Moreover, protologues for similar species were also consulted.

## Taxonomic treatment

### Argyreia pseudosolanum

Taxon classificationPlantaeSolanalesConvolvulaceae

Traiperm & Suddee sp. nov.

45928FA3-6E81-549B-8506-8131FF35B4B7

urn:lsid:ipni.org:names:77209855-1

**Figure 1.**
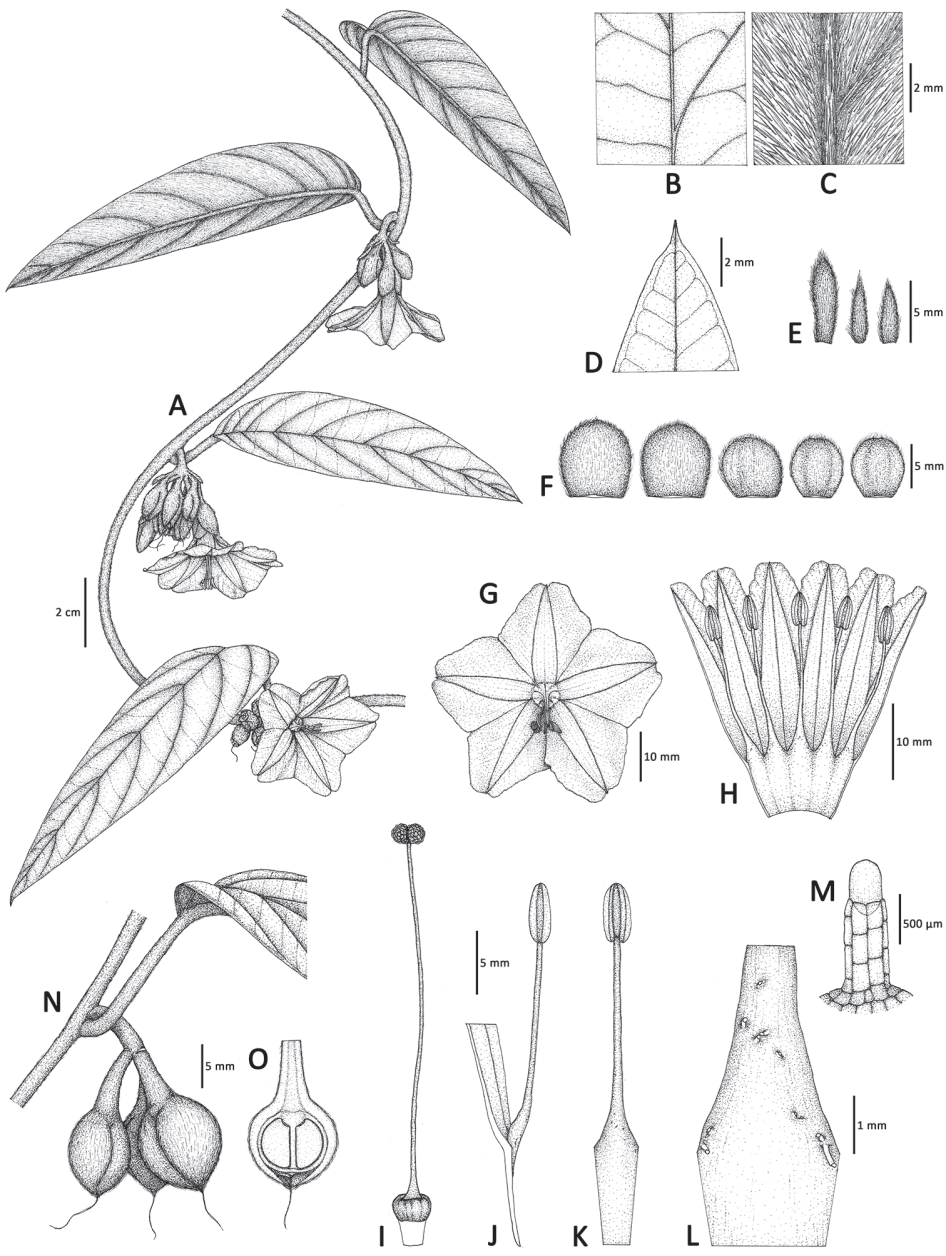
*Argyreia pseudosolanum*. **A** Stem with leaves and inflorescences **B** adaxial leaf surface **C** abaxial leaf surface **D** upper part of leaf, showing secondary veins on adaxial leaf surface **E** inflorescence bracts, outer (left) to inner (right) **F** 5 sepals from outer (left) to innermost (right) **G** flower in front view **H** opened corolla with 5 stamens **I** pistil, showing undulate disc and biglobose stigma **J** filament insertion showing an attachment point **K** single stamen **L** close-up of lower part of stamen, showing a few multicellular uniseriate hairs **M** multicellular uniseriate hair **N** young fruits with sepals **O** fruit in longitudinal section, showing 2 immature seeds. All drawn by N. Chitchak from voucher specimens *Suddee et al. 5363* (BKF) (**A–M**), *P. Kochaiphat 353* (BKF) (**N, O**).

**Figure 2.**
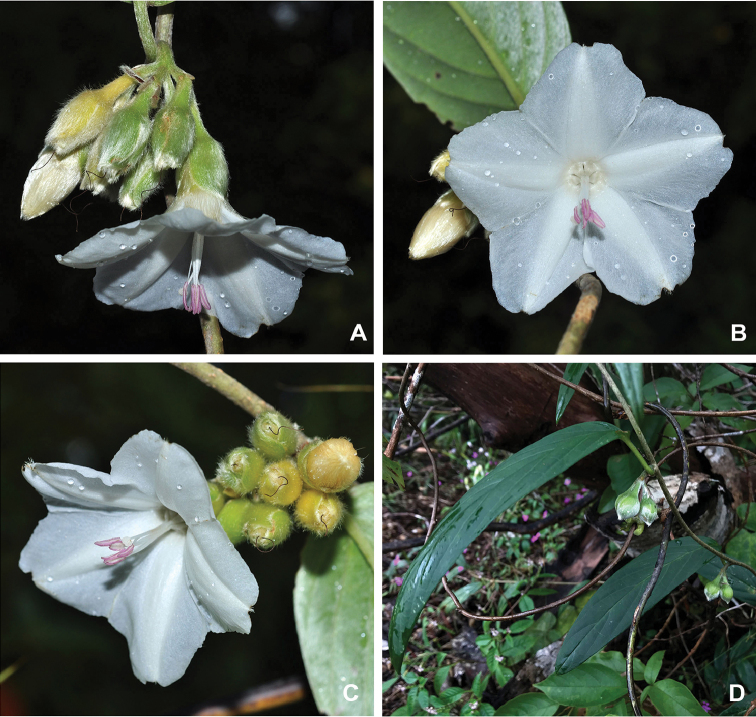
*Argyreia pseudosolanum* inflorescence, corolla and young fruit details. **A** and **C** inflorescence and flower in lateral view, showing cymose inflorescence with short peduncle, and rotate corolla shape **B** flower in frontal view, showing 5-lobed corolla limb (star-shaped), exserted genitalia and pinkish anthers (voucher: *Suddee et al. 5363*) **D** young fruits (voucher: *P. Kochaiphat 353*). Photographs **A–C** by W. Kiewbang, **D** by P. Kochaiphat.

Figs 1 , 2 , Table 1

#### Diagnosis.

Similar to *Argyreia corneri* in having a white rotate corolla, but differs in narrowly elliptic, oblong or lanceolate leaf shape (versus ovate), the ovate outer sepal shape (versus broadly ovate), the limb distinctly star-shaped with 5-triangular lobes (versus limb vaguely 5-angled).

#### Type.

Thailand, Bueng Kan Province, Bueng Khong Long District, Phu Langka National Park, 530 m elev., 12 Sep 2018, *S. Suddee, P. Puudjaa, C. Hemrat & W. Kiewbang 5363* (holotype: BKF!; isotypes: BKF!, K!, QBG!).

#### Description.

Climbers up to 2 m tall; stems woody at base, herbaceous above, 1.5–3 mm diam. on flowering stems; covered at first with simple, non-glandular, appressed greyish trichomes, later glabrous or glabrescent, grooved lengthwise when dry. Leaves simple; petioles 0.3–1.2 cm long, grooved adaxially, densely-appressed whitish hairy or glabrescent; leaf blades narrowly elliptic, oblong or lanceolate, 7–17 × 1–5 cm, base cuneate to obtuse or rounded, margins entire, apex acute or mucronate-apiculate; papery or chartaceous, adaxially dark green, glabrous, abaxially paler, densely-appressed shining sericeous; secondary veins 5–8 on either side, veins slightly raised adaxially, more prominently raised abaxially. Inflorescences axillary cymes, 3–5-flowered; peduncle cylindrical, 4–6 mm long, densely-appressed sericeous; bracts narrowly lanceolate, 5–7 × 1.5–2 mm, adaxially glabrous, abaxially densely whitish hairy, caducous; pedicels 4–5 mm long, terete, whitish sericeous. Flowers diurnal; sepals subequal, 2 outer obovate-elliptic, 7.5–8 × 6–8 mm, abaxially whitish sericeous, adaxially glabrous, margins entire, apex rounded, third sepal asymmetrical, 2 inner sepals obovate-rounded, 5–6 × 6–7 mm, abaxially whitish sericeous, adaxially glabrous, thinner at margins. Corolla rotate, 1.8–2 cm long, pure white, tube short, limb distinctly star-shaped, ca. 3 cm in diam., 5-lobed, lobes triangular, apex emarginate; densely appressed whitish sericeous outside on mid-petaline bands and tube. Stamens exserted, subequal, 18–24 mm; filaments basally fused to corolla tube, glabrous or nearly glabrous with a few multicellular uniseriate hairs at attachment point; anthers oblong-sagittate, 3–4 mm long, longitudinally dehiscent. Pistil exserted, slightly longer than stamens, white; disc annular, undulate; style simple, filiform, glabrous, base expanded, conical; stigmas 2, subglobose with flattened base to globose. Fruit berries, globose to subglobose, enclosed in enlarged calyx, 0.8–1 cm in diam.

#### Phenology.

Flowering and fruiting (young fruit) in September, during the latter part of the rainy season.

#### Distribution and ecology.

In mixed deciduous forest on a sandstone plateau. Elevation: 530 m.

#### Vernacular name.

Khruea sawate phulangka (เครือเศวตภูลังกา), the name is given by the authors.

#### Etymology.

The specific epithet refers to the corolla shape, which is similar to Solanaceae and not found elsewhere in *Argyreia*.

#### Conservation status.

*Argyreia pseudosolanum* is known only from Phu Langka National Park with an estimated area of occupancy around 1 km^2^. The number of mature individuals in the population is less than 50. The species occurs near the large golden stupa on the top of the Phu Langka plateau which has religious activities in the dry season. This area might be disturbed by these activities and this could affect the survival chances of this species. It is assessed here as Critically Endangered, CR B2ab(iii); D, following the IUCN Criteria (2017).

#### Additional specimens examined.

Thailand, Bueng Kan Province, Bueng Khong Long District, Phu Langka National Park, 530 m elev., 12 Sep 2018, *S. Suddee, P. Puudjaa, C. Hemrat & W. Kiewbang 5363* (BKF!, K!, QBG!); 27 Sep 2018, *P. Kochaiphat 353* (BKF! 2sht., K!, QBG!).

**Table 1.** Main characters distinguishing *Argyreia pseudosolanum* from its morphologically similar species in Thailand, *A. fulvocymosa* C.Y.Wu (Staples and Traiperm 2010) and in Peninsular Malaysia, *A. corneri* Hoogland (Staples and Syahida-Emiza 2015).

Character	*A. corneri*	*A. fulvocymosa*	*A. pseudosolanum*
Leaf shape and size	ovate, 3.5–9 cm × 2–5 cm	broadly ovate-circular to nearly circular, 12–16.5 cm × 10–15 cm	narrowly elliptic, oblong or lanceolate, 7–17 cm × 1–5 cm
Bract shape and size	linear, ca. 10 mm long, caducous	early deciduous	narrowly lanceolate, 5–7 mm × 1.5–2 mm, caducous
Sepals proportions	unequal, 3 outer larger: 2 inner smaller	unequal, 2 outer larger: 3 inner smaller	subequal, 2 outer larger: 3 inner smaller
Outer sepal shape and size	broadly ovate, ca. 8.5 mm long	broadly ovate-circular, ca. 5 × 4 mm	obovate-elliptic, 7.5–8 mm × 6–8 mm
Corolla shape	rotate, ca. 2.5–3 cm long, limb vaguely 5-angled	funnelform, ca. 2 cm, limb distinctly 5-lobed	rotate, 1.8–2 cm long, limb distinctly star-shaped, 5-triangular lobes
Stamen and pistil	exserted	exserted	exserted
Distribution	Peninsular Malaysia (Pahang), not found in Thailand	S China to Indo-China, N Thailand: Phitsanulok Province	endemic to NE Thailand: Bueng Kan Province

## Supplementary Material

XML Treatment for Argyreia pseudosolanum
